# Parenting Desire and Minority Stress in Lesbians and Gay Men: A Mediation Framework

**DOI:** 10.3390/ijerph15102318

**Published:** 2018-10-22

**Authors:** Anna Lisa Amodeo, Concetta Esposito, Vincenzo Bochicchio, Paolo Valerio, Roberto Vitelli, Dario Bacchini, Cristiano Scandurra

**Affiliations:** 1 Department of Humanistic Studies, University of Naples Federico II, 80138 Napoli, Italy; amodeo@unina.it (A.L.A.); dario.bacchini@unina.it (D.B.); 2 SInAPSi Center, University of Naples Federico II, 80138 Napoli, Italy; concetta.esposito3@unina.it; 3 Department of Humanistic Studies, University of Calabria, 87036 Rende, Italy; vincenzo.bochicchio@unical.it; 4 Department of Neurosciences and Reproductive and Odontostomatological Sciences, University of Naples Federico II, 80138 Napoli, Italy; valerio@unina.it (P.V.); rvitelli@unina.it (R.V.); cristiano.scandurra@unina.it (C.S.)

**Keywords:** parenting desire, lesbian, gay, minority stress, mediation

## Abstract

Despite the rapid increase in lesbian and gay (LG) people who desire and decide to become parents, LG childless individuals may encounter serious obstacles in the parenthood process, such as minority stress. Notwithstanding, the psychological processes by which prejudice events might affect the desire to become parents are still understudied. As an extension of the minority stress theory, the psychological mediation framework sheds light on these psychological processes, as it encompasses a more clinical view of stress. Within this framework, the current study aimed at assessing the role of prejudice events in affecting parenting desire in 290 childless Italian LG individuals (120 lesbians and 170 gay men), as well as the role of internalized heterosexism and sexual orientation concealment in mediating the relationship between prejudice events and parenting desire. The results suggest that only in lesbians prejudice events were negatively associated with parenting desire, and that sexual orientation concealment and internalized heterosexism were also negatively associated with parenting desire. Furthermore, sexual orientation concealment, and not internalized heterosexism, mediated the relationship between prejudice events and parenting desire in lesbians, but not gay men. The findings have important implications for clinical practice.

## 1. Introduction

Becoming parents represents a complex process that might be influenced by many social dimensions, such as economic issues, familial policies, local legislation, or housing conditions [1]. From a psychological point of view, a starting point in the parenthood path is the desire to become a parent, or rather “what one wants or would like to do” [2] (p. 10).

Despite the rapid increase in lesbian and gay (LG) people who desire and decide to become parents, differently from heterosexual people, LG childless individuals may encounter serious obstacles in the parenthood process due to their sexual minority status [3,4,5]. One of these is represented by minority stress, a specific stress caused by socially stigmatized status and associated with negative health outcomes [6,7]. Minority stress theory (MST) conceptualizes both distal and proximal stressors. Distal stressors are objective stressors (i.e., prejudice events, such as interpersonal violence, employment discrimination, mistreatment in healthcare setting, etc.), that are independent of the individual’s perceptions or feelings. On the other hand, proximal stressors are subjective and internal stressors dependent on individual’s perceptions, such as expectations of rejection, sexual orientation concealment, and internalized heterosexism, that is, the direction of negative social attitudes toward the self. MST assumes that both stressors assumes that objective stressors (i.e., prejudice events, such as violence and discrimination), as well as subjective stressors (i.e., expectations of rejection, sexual orientation concealment, and internalized homophobia), predict negative health outcomes, and that specific protective factors (e.g., resilience, community connectedness, social support, etc.) buffer this direct association [7,8]. Recently, some studies have demonstrated that minority stressors negatively affect parenting desire, thus resulting in a significant psychosocial barrier to parenthood [9,10,11].

A recent extension of MST—the psychological mediation framework (PMF)—places the accent on a more clinical and subjective view of the stress, thanks to the use of proximal stressors as mediators between prejudice events and health [12]. The PMF was postulated to analyze psychological paths linking stigma-related stressors to negative health outcomes. Thus, differently from MST, according to which stress is a mediator between social structure/status and health, the PMF looks at stress as the starting point of a mediating psychological chain leading to negative health outcomes. Psychological mediators are both the proximal minority stressors (e.g., internalized heterosexism and sexual orientation concealment) and some general psychological processes (e.g., interpersonal problems or emotion dysregulation). In summary, if MST considers both distal and proximal minority stressors as predictors of health outcomes, the PMF considers the proximal minority stressors as mediators between distal minority stressors and health.

Although recent studies have demonstrated the usefulness of approaching parenting dimensions as potential outcomes of the minority stress processes, going beyond the health outcomes [9,10,11], no previous studies have applied the PMF to parenting dimensions in LG individuals. As parenting desire represents the first link in the chain of the parenting process, and internalized heterosexism and sexual orientation concealment are the two main personal issues shaping parenting decisions [13], we were interested in analyzing the role of internalized heterosexism and sexual orientation concealment as mediators between prejudice events and parenting desire in childless LG individuals.

Furthermore, as gender is a fundamental dimension of parenthood [14], the difference between women and men was considered as a potential moderator. Indeed, although the old sexist stereotypes have been overcome in contemporary society, some different-sex couples still divide their childcare tasks or household chores based on gender norms, with women dedicated to these tasks and men employed outside the home [15]. On the contrary, most of the research on same-sex couples reported that they are more likely than different-sex couples to divide the labor fairly [16]. These generalizations must not lead to the perception of same-sex couples as more functioning than different-sex couples, but serve to show that gender might play a key role in the couple’s dynamics and that it should be considered as a crucial factor in studies exploring parenthood dimensions. The article begins by providing an overview of the relationship between minority stressors and parenting desire in LG individuals. Then, it gives an overview of the internalized heterosexism and sexual orientation concealment as mediators, according to the PMF. Finally, as Italy represents the context of our study, it introduces a snapshot of the Italian context for LG individuals.

### 1.1. Minority Stressors and Parenting Desire in Lesbians and Gay Men

LG individuals have been found to be generally resilient in the face of stigma and to resist heterosexist social pressure [17,18]. In spite of this, they still experience prejudice events due to their stigmatized social status, thus experiencing high rates of minority stress and negative health outcomes [17,18]. When childless LG individuals become parents, they also become extremely visible and, because of this, minority stress might increase [11].

Recent studies have shown the association between minority stress and parenting dimensions. For instance, Bos et al. [10] found that lesbian mothers experiencing higher levels of prejudice events showed more parental stress and, at the same time, felt more pressed to justify their motherhood qualities to people than mothers experiencing fewer prejudice events. Similarly, mothers with higher levels of internalized heterosexism tended to defend their position as mothers more often than those with lower levels of internalized heterosexism. Another study, by Baiocco et al. [9], found that childless Italian LG adults with higher levels of internalized heterosexism were less likely to desire to marry, and to recognize some positive effects of same-sex legal recognition, than those with lower levels of internalized heterosexism. More recently, an Italian study by Scandurra et al. [11], in which MST was applied to a group of childless LG individuals, reported that prejudice events, sexual orientation concealment, and internalized heterosexism were negatively associated with parenting desire in lesbians, and that felt stigma negatively impacted parenting desire in gay men. Furthermore, support from family or significant others buffered the relationship between minority stressors and parenting desire.

These data seem to confirm that one of the obstacles that LG individuals may encounter in their parenthood path is heterosexism [4,5], which, in turn, leads to experiencing minority stress because sexual minority people do not match heterosexist expectations. Indeed, as suggested by Mezey [13], due to the heterosexist society in which LG individuals live, two fundamental personal and psychological dimensions that have to be considered in analyzing the parenthood process are internalized heterosexism and sexual orientation concealment.

LG individuals internalize societal values and messages that communicate that nonheterosexual orientations are immoral, that LG individuals cannot be good parents, and that children born to a same-sex couple will not grow up well [4,19,20]. Due to internalized heterosexism, childless LG individuals may come to question their ability to become parents, creating barriers to parenthood [13]. Sexual orientation concealment, which is the negative side of the coming out process, is also connected to internalized heterosexism. Revealing one’s own nonheterosexual orientation to family members, friends, and colleagues, may have positive effects on the quality of relationships and mental health [21,22]. At the same time, it is often a difficult and pained process, which depends on several factors, such as age, class, race, and environment [19]. Indeed, this process implies the necessity to negotiate multiple identities in one’s own environment, and this is particularly true for LG individuals who desire or want to become parents [13]. For instance, the fear that some LG individuals experience in coming out to their parents might represent a serious obstacle to the parenthood process [23]. Finally, despite similarities in the coming out process among lesbians and gay men, previous studies [24,25] have detected an interesting difference based on gender: many lesbians tend to perceive coming out as a necessary step to becoming mothers, while many gay men tend to regard coming out as a barrier to becoming fathers. Thus, for some gay men, coming out represents a break between being gay or being a father [26]. Substantially, it seems that this gender difference might be explained through the social expectations regarding becoming a parent, which are greater for women than for men [27]. For this reason, the individual’s gender should be considered as a potential moderator in parenting studies.

### 1.2. Internalized Heterosexism and Sexual Orientation Concealment According to the Psychological Mediation Framework

As mentioned above, starting from a clinical view of stress and with the aim of extending the MST, the PMF was postulated as a theoretical framework to better understand psychological pathways that link minority stressors to negative health outcomes. Previous studies have empirically supported the validity of the PMF, both in LG [28,29] and transgender and gender nonconforming [30,31,32] people.

Specifically, and considering the main dimensions of the current study, there is evidence that internalized heterosexism and sexual orientation concealment mediate the relationship between prejudice events and health. For instance, Feinstein et al. [33] found that internalized heterosexism mediated the association between experiences of discrimination and both depression and social anxiety in both lesbians and gay men.

On the other hand, Ryan et al. [34] found that negative reactions to disclosure, which might be viewed as a form of prejudice event, were associated with depression and low self-esteem, and that autonomy need satisfaction following disclosure was a mediator between negative reactions and health. It is noteworthy that sexual orientation disclosure is not always beneficial, both because LG individuals could experience negative reactions from their interpersonal contexts and due to the inherently stressful process [35,36].

To our knowledge, the PMF has not yet been applied to parenting dimensions (e.g., parenting desire), although previous studies have considered these dimensions as potential outcomes of MST [9,10,11].

### 1.3. Italian Context for Lesbians and Gay Men

The Italian government has only recently recognized same-sex civil unions (law No. 76/2016), specifically in June 2016, and only after a scathing battle between conservative parties, supported by strongly religious groups, and moderate and left-wing parties. Before final approval of the original law, which foresaw the same benefits of different-sex marriage to same-sex couples, the so-called stepchild adoption (i.e., the possibility of adopting the biological child of a partner) was removed. This means that the only public body that can decide in favor of the adoption of a partner’s biological child is the Supreme Court of Appeal, which has already provided this possibility to some Italian same-sex couples. Notwithstanding, contrary to different-sex couples, Italian same-sex couples cannot legally adopt children.

It is noteworthy that the social and political debate that led to law No. 76/2016 was specifically around the parental abilities of LG people, who were perceived by the opposition parties to be inadequate parents [11]. Along the same lines, a recent study by Lasio et al. [37], analyzing the speeches of Parliamentarians who expressed their opposition to LG parenthood, revealed that discourses were organized around a hegemonic model of gender, and that they contributed to reiterating old models of motherhood, maintaining the institutionalization of sexuality and reproduction within a patriarchal logic. These findings seem to be supported by some recent Italian studies, in which it has been reported that Italy is a strongly heteronormative [20,38,39,40,41,42] and genderist [43,44] society that still tends to equate women to mothers. Thus, social status differences continue to exist in Italy, although a great amount of progress has been made in the last few years.

Social status difference represents the most important dimension that leads to the development of minority stress, as it highlights the existence of social inequalities that, in turn, might be internalized by minority groups as a distinctive sign of their own identity. Indeed, previous studies underlined the presence of high levels of internalized heterosexism in Italian LG population [39], as well as in LG individuals who were in the reproductive age [9]. Unfortunately, despite the positive legal progress, Italian LG individuals still experience high rates of minority stressors [45], which, in turn, negatively affect parenting desire [11].

### 1.4. The Current Study

This study was aimed at filling a gap in the literature by applying the PMF to parenting desire in childless Italian LG individuals. Previous studies have already demonstrated that MST is a useful perspective for understanding psychosocial obstacles to parenting desire [9,10,11]. On the contrary, to our knowledge, although the PMF is an extension of MST, no previous studies have applied this framework to parenting desire.

Informed by MST, we first hypothesized that prejudice events could be negatively associated with parenting desire and, based on previous Italian studies highlighting the heteronormative social context for Italian LG individuals [20,38,39,40,41,42], that this association might be stronger in childless lesbian women than in childless gay men (Hypothesis 1). Second, informed both by the PMF and previous Italian studies [20,38,39,40,41,42], we hypothesized that the most subjective minority stressors (i.e., sexual orientation concealment and internalized heterosexism) would be negatively linked to parenting desire, with a stronger effect in lesbian women (Hypothesis 2). Third, informed by the PMF, we expected that both sexual orientation concealment and internalized heterosexism would act as mediators between prejudice events and parenting desire, and that these indirect effects would be dependent on an individual’s gender (woman vs. man) (Hypothesis 3). Finally, as sociodemographic factors might influence both minority stressors and parenting desire [3,11,46,47], we considered the possible confounding effect of the following dimensions, age, education level, political orientation, and having a partner.

## 2. Materials and Methods

### 2.1. Participants

A total of 290 childless LG individuals (120 lesbians and 170 gay men), recruited online, participated in the study. The total sample ranged in age from 18 to 50 years (lesbians, M = 28.25, SD = 6.14; gay men, M = 33.11, SD = 9.64). Participants could participate in the online survey only if they self-identified with lesbian or gay sexual orientation, were of the Italian age of consent (i.e., 18-years old), did not already have children, and had lived in Italy for at least 10 years. Nineteen participants were excluded due to uncompleted questionnaires. Demographic characteristics for both the total sample and the sample differentiated by gender are reported in Table 1.

### 2.2. Procedures

Participants answered the questionnaires at the end of 2016, when the law on same-sex civil unions was yet to be approved. The survey was disseminated through the main social networks (e.g., Facebook), posting the advertisement on many LG groups. Furthermore, some Italian non-governmental organization (NGO) representatives involved in the defense of lesbian, gay, bisexual, transgender, and queer rights helped us to carry out a snowball sampling recruitment, asking their affiliates to answer the questionnaire and to disseminate the survey to their personal contacts. No rewards were provided to participate in the survey.

The study was designed with regard to all of the principles of the Declaration of Helsinki on Ethical Principles for Medical Research Involving Human Subjects. According to the EU Regulation 2016/679 “General Data Protection Regulation” (GDPR) and the Code of Conduct for Research in Psychology of the Italian Association of Psychology approved on 27th March 2015, the participants gave their informed consent before completing the questionnaire. Furthermore, in accordance with Italian legislation, privacy was guaranteed through a secure gateway, accessible only to the Principal Investigator, who removed all of the IP addresses before sharing any data with the other researchers.

### 2.3. Measures

#### 2.3.1. Sociodemographic Characteristics

Sociodemographic variables included gender (male, female, other with specification required), sexual orientation, age, level of education (≤high-school and ≥college), political orientation (left-wing, centrist, right-wing), and actual stable partner (yes/no).

#### 2.3.2. Parenting Desire

A single-item question based on the work of Baiocco and Laghi [48] was used to determine information about parenting desire: “Would you like to have a baby?”. The response options were dichotomous (yes/no). In the current sample, 95 (79.2%) lesbians and 123 (72.4%) gay men responded positively to the question.

#### 2.3.3. Prejudice Events

The Experiences of Discrimination Scale (EDS) [49,50] is an eight-item measure for assessing four prejudice events: verbal abuse (e.g., “I heard jokes or unpleasant or derogatory comments about my sexual orientation”), unequal treatment (e.g., “Because of my sexual orientation I have not been able to get something important to me, for example a grant, a job”), avoidance (e.g., “It happened to me that some people have avoided me because of my sexual orientation”), and victimization (e.g., “I was physically assaulted because of my sexual orientation”). The response options ranged from “never” to “often” on a five-point Likert scale. Higher scores indicate more prejudice events. The internal consistency reliability was α = 0.84.

#### 2.3.4. Internalized Heterosexism

The Measure of Internalized Sexual Stigma for Lesbians and Gay Men (MISS-LG) [39] is a 17-item questionnaire that assesses the negative attitudes of LG people towards homosexuality and specific aspects of themselves. A final score is achieved through three dimensions: (1) identity, or rather the enduring propensity to have negative self-attitudes (e.g., “I’d prefer to be heterosexual”); (2) social discomfort, that is the fear of publicly identifying as a LG individual, disclosure at work and in one’s personal life, and negative beliefs about the religious, political, and moral acceptability of nonheterosexual orientation (e.g., “I’m careful of what I wear and what I say to avoid showing my homosexuality”); and (3) sexuality, or rather negative attitudes towards other LG individuals, and negative evaluation of same-sex relationships and sexual behaviors in LG people (e.g., “I don’t believe in love between homosexuals”). The response options ranged from “I disagree” to “I agree” on a five-point Likert scale. The internal consistency reliability of the entire scale was α = 0.80 for the lesbian form and α = 0.81 for the gay man form. We used the total score of the scale, as done by other authors [51]. Higher scores indicate greater internalized heterosexism.

#### 2.3.5. Sexual Orientation Concealment

The Outness Inventory (OI) [39,52] is an 11-item scale for assessing the degree of openness about an LG individual’s sexual orientation with 11 individuals or groups (i.e., mother, father, siblings, extended family, new and old straight friends, work peers and supervisors, members and leaders of religious communities, and strangers). This measure included three scales: family, world, and religion. Overall outness was calculated as the subscales average. The response options ranged from “person definitely does not know about your sexual orientation status” to “person definitely knows about your sexual orientation status, and it is openly talked about” on a seven-point Likert scale. Scores were recoded so that higher scores indicated greater sexual orientation concealment. In the current study, the internal consistency reliability for the entire scale was α = 0.80.

### 2.4. Statistical Analyses

Analyses were carried out using IBM SPSS statistics version 21 (IBM Corp., Armonk, New York, NY, USA). The PROCESS macro version 3.1 [53] was used to test the study’s hypothesized model, which included both a moderation (i.e., the individual’s gender) and a mediation (i.e., sexual orientation concealment and internalized heterosexism) component.

**Figure 1:**
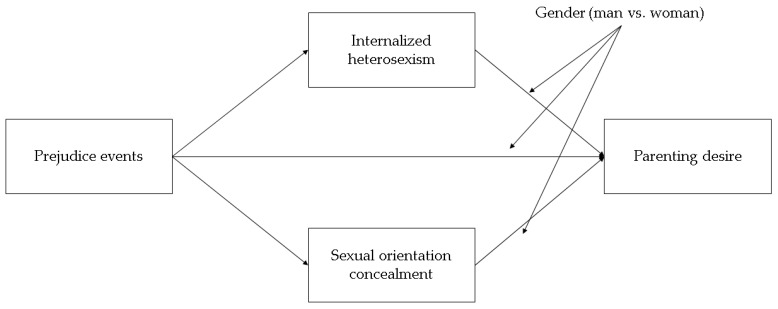
The hypothesized moderated mediation model. For reasons of simplification, control variables were not reported in the figure.

A total effect moderation model (model template 59 in the PROCESS macro) was first specified. In this model, the effect of prejudice events (independent variable) on parenting desire (binary dependent variable; direct effect), and on sexual orientation concealment and internalized heterosexism (mediators), as well as the effect of both sexual orientation concealment and internalized heterosexism on parenting desire, were supposed to be conditional (or moderated) by the individual’s gender (woman vs. man); however, estimation of the coefficients in the statistical model revealed no significant interaction between prejudice events and gender on both sexual orientation concealment and internalized heterosexism (both *ps* > 0.05). Since nonsignificant interactions also influenced the estimate of the hypothesized indirect effects (which were necessarily conditional with these interactions in the model), along with all inferential tests thereof, they were trimmed in the final moderated mediation model (Figure 1), and were thus expressed by only three interaction terms: (1) gender by prejudice event interaction on parenting desire; (2) gender by sexual orientation concealment interaction on parenting desire; and (3) gender by internalized heterosexism interaction on parenting desire (model template 15 in PROCESS macro).

Significant conditional direct and indirect effects were probed by using the pick-a-point approach. As a final step, in order to quantify the indirect effects as a function of the moderator, and to provide inferential tests for those conditional indirect effects, indices of moderated mediation were estimated for each of the hypothesized moderated mediation paths, as recommended by Hayes [53]. Bias-corrected bootstrap confidence intervals, based on 5000 resamples, were used as indicators of effect size. Confidence intervals that did not contain zero indicated a significant indirect effect via the specific mediator.

## 3. Results

### 3.1. Descriptive Statistics and Preliminary Analyses

The means, standard deviations, and bivariate correlations between all variables, separately for lesbians and gay men, are reported in Table 2. The results highlighted a significant positive association only between parenting desire and internalized heterosexism in lesbians; no significant associations resulted for gay men. Age was negatively correlated with internalized heterosexism, and positively associated with sexual orientation concealment, in both LGs. Having a stable partner was associated with internalized heterosexism in lesbians, whilst sexual orientation concealment was associated with the absence of a stable partner in both samples.

As a further preliminary step, we examined gender differences on prejudice events, sexual orientation concealment, and internalized heterosexism by performing a multivariate analysis of variance, including age, education, political orientation, and stable partner as covariates (MANCOVA). The analysis showed significant differences between lesbians and gay men, with Wilks’ lambda = 0.92, *p* < 0.001, F (3282) = 8.37, η^2^ = 0.08. Specifically, gay men reported higher levels of prejudice events—F (1284) = 16.59, *p* < 0.001, η^2^ = 0.06—and internalized heterosexism—F (1284) = 4.20, *p* < 0.05, η^2^ = 0.01—than lesbians did. No significant difference was found based on sexual orientation concealment.

### 3.2. Associations between Minority Stressors and Parenting Desire

The results of the regression analysis are presented in Table 3.

**Figure 2:**
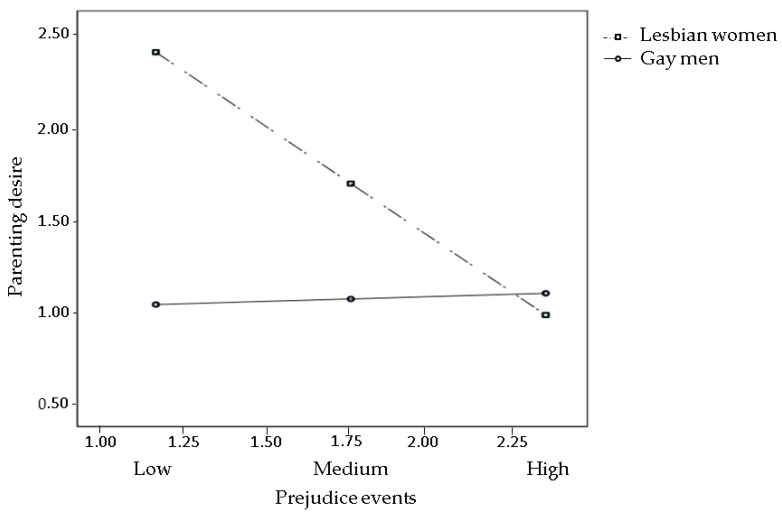
Interaction between prejudice events and gender on parenting desire. Estimated log odds of parenting desire are reported for combinations of prejudice events (low, medium, and high) and individual’s gender (lesbian women vs. gay men).

**Figure 3:**
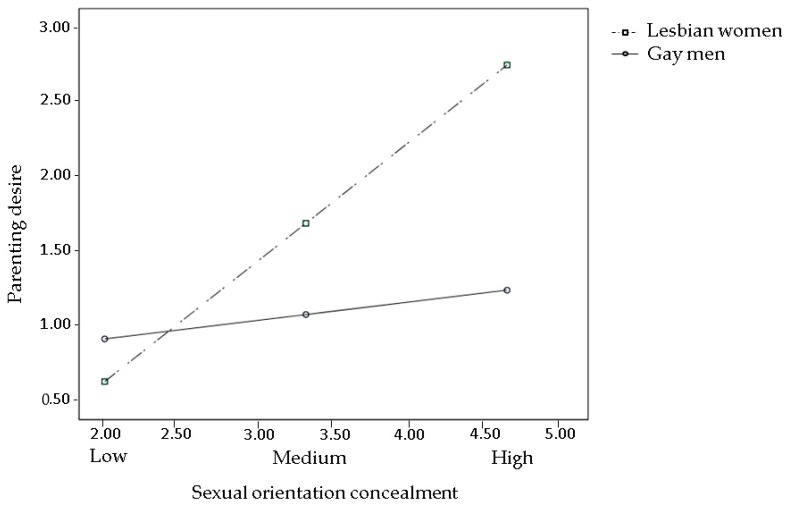
Interaction between sexual orientation concealment and gender on parenting desire. Estimated log odds of parenting desire are reported for combinations of sexual orientation concealment (low, medium, and high) and individual’s gender (lesbian women vs. gay men).

**Figure 4:**
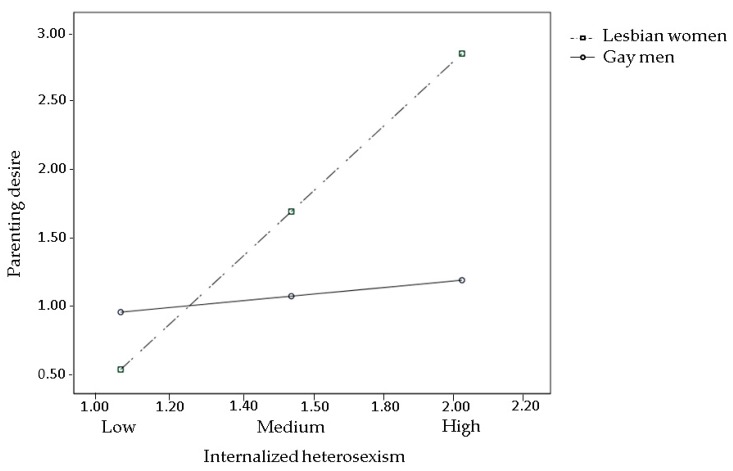
Interaction between internalized heterosexism and gender on parenting desire. Estimated log odds of parenting desire are reported for combinations of internalized heterosexism (low, medium, and high) and individual’s gender (lesbian women vs. gay men).

The effects of prejudice events (Hypothesis 1), sexual orientation concealment, and internalized heterosexism (Hypothesis 2) on parenting desire were all moderated by gender (all interaction terms were significant). The pick-a-point approach revealed that prejudice events significantly reduced parenting desire in lesbians, but not in gay men (Figure 2), with Bs = −1.18 and 0.05, *ps* = 0.01 and 0.86, 95% CIs (−2.08, −0.28) and (−0.52, 0.63), respectively. Both sexual orientation concealment and internalized heterosexism were positively associated with parenting desire in lesbians, but not in gay men (Figure 3 and Figure 4, respectively), with Bs = 0.81 and 0.12, *ps* = 0.01 and 0.36, 95% CIs (0.20, 1.42) and (−0.14, 0.39) for sexual orientation concealment, respectively; and Bs = 2.41 and 0.24, *ps* = 0.003 and 0.55, 95% CIs (0.78, 4.04) and (−0.55, 1.04) for internalized heterosexism, respectively.

With respect to moderated mediation effects (Hypothesis 3), we found only one indirect effect of prejudice events on parenting desire through sexual orientation concealment, which involved lesbians, but not gay men. The index of moderated mediation confirmed the significance of this effect (Table 4).

### 3.3. Control Variables

Only age and having a stable partner were found to significantly predict outcomes in our study. Age was negatively associated with internalized heterosexism and parenting desire, whereas it was positively linked to sexual orientation concealment. Having a stable partner was associated with higher internalized heterosexism and lower sexual orientation concealment. Furthermore, having a stable partner was negatively associated with parenting desire.

## 4. Discussion

Informed by previous studies that used MST to explore parenting dimensions in LG individuals [9,10,11], as well as those that highlighted the heteronormative context for Italian LG individuals [20,38,39,40,41,42], the current study intended to apply part of the PMF as an extension of MST to parenting desire to a group of childless Italian LG individuals. We found partial support for this operation. Indeed, our results highlighted both a direct and an indirect effect (through sexual orientation concealment) of prejudice events on parenting desire, but only in lesbians. Of note, the direction of this effect changed depending on whether the association was direct or indirect. Specifically, we found that prejudice events were negatively associated with parenting desire, but that this effect became positive when it was mediated by sexual orientation concealment. Thus, our findings shed light on clinical practice, highlighting the need for structuring clinical interventions to ameliorate the negative effects of minority stressors on parenting desire.

In support of the first hypothesis of this study, we found that prejudice events were negatively associated with parenting desire only in lesbians and not in gay men. This means that, unlike gay men, prejudice events reduce the likelihood of lesbians desiring to become mothers. This finding might be read through the lens of MST, as well as through evidence that the Italian context is a highly heteronormative social context. To this end, previous studies have already demonstrated that discrimination episodes negatively impact parenting desire in lesbians, but not in gay men [11], and that this gender difference is due to the double stigma that many lesbians experience as both lesbians and women [54]. Furthermore, experiencing a prejudice event due to one’s own sexual orientation and/or gender identity ends up emphasizing a status difference, as what is affected is a specific identity dimension. It is then likely that, in a heteronormative context, such as the Italian one [20,38,39,40,41,42], a lesbian woman experiencing a prejudice event will perceive this difference in status more than a gay man, because parenthood seems to still be a woman’s prerogative [9,11]. Thus, a lesbian woman might feel herself to be less adequate than a gay man to become a parent, and this ends up decreasing the levels of parenting desire.

Regarding the second hypothesis, that sexual orientation concealment and internalized heterosexism negatively affect parenting desire in lesbians, but not in gay men, our findings were significant but, contrary to our expectations, they suggested that both subjective minority stressors were positively associated with parenting desire in lesbians and not in gay men, increasing the level of parenting desire in lesbians, rather than decreasing it. This unexpected finding might be read through the lens of MST and the related social pressure that some minority people could feel to conform to social expectations. Thus, it is probably that higher levels of internalized heterosexism, in addition to questioning one’s own parenting abilities [13], might lead to a need to conform to social expectations, including becoming a mother in a context that still promotes motherhood as a key value for female identity [48]. Similarly, in line with the finding discussed above—that internalized heterosexism increases parenting desire in lesbians—the fact that sexual orientation concealment increased parenting desire only in lesbians, and not in gay men, might be read through the hypothesis that concealing one’s own sexual orientation would lead lesbians to conform to societal expectations that tend to equate women to mothers. Notwithstanding these interpretative hypotheses, this finding should be qualitatively investigated to better understand how internalized stigma and sexual orientation concealment could lead to an increase in parenting desire.

Finally, regarding the third hypothesis (the moderated mediation model) that both sexual orientation concealment and internalized heterosexism would mediate the relationship between prejudice events and parenting desire, and that gender would moderate these indirect effects, our findings provided partial support. Indeed, only sexual orientation concealment, and not internalized heterosexism, acted as a significant mediator between prejudice events and parenting desire, and only in lesbians, increasing, rather than decreasing, their level of parenting desire. Thus, in the presence of sexual orientation concealment, prejudice events ended up increasing parenting desire in lesbians. As suggested by previous studies [24,25], unlike in gay men, the visibility of lesbian motherhood in previous decades, together with the social expectations that a woman will become a mother [27], might lead lesbians to perceive coming out as a mandatory step to becoming mothers. Thus, as suggested by our findings, experiencing prejudice events as a lesbian woman might lead to concealing one’s own sexual orientation, which, in turn, increases the desire to become a mother, as becoming a mother would increase one’s visibility as a normative woman, fitting the heteronormative equivalence of women as mothers.

### 4.1. Limitations and Suggestions for Future Research

This study had significant limitations that might affect the generalizability of the results to the general Italian LG population. First, the cross-sectional design of the study did not allow certain inferences to be made concerning the causal relationships between variables. Indeed, findings have to be interpreted as associations which do not necessarily prove causality between variables. Future longitudinal studies should assess the cause-and-effect relationships between minority stressors and parenting desire, analyzing potential changes over time.

A second limitation concerns the use of a single-item question to assess parenting desire. Although the same question had been used in a previous study [46], a more composite measure on parenting desire should be used in future research. Similarly, a qualitative investigation on parenting desire, and on its relationship with minority stressors, is recommended in order to achieve a better understanding of the psychological processes underlying these dimensions.

A third limitation was due to a sample selection bias. Indeed, participants were mostly recruited through social networks and Italian NGOs engaged in the defense of LG rights, thus not representing the general LG population. This may even explain why no right-wing individuals participated in the survey. Finally, in line with the previous limit, a last limitation was the absence of diverse ethnic groups of LG individuals, which prevented us from reading the findings from an intersectionality perspective. To this end, it is plausible to hypothesize that sociocultural differences between Caucasians and non-Caucasians living in Italy exist, both in terms of minority stress—that is usually more pervasive in multiple marginalized individuals [55]—and parenting process.

### 4.2. Implications for Clinical Practice

Clinicians might encounter LG individuals for many reasons, including LG individuals or couples who want to become parents or are questioning about a parenting plan. The results of this study might provide helpful suggestions for clinical practice with childless LG individuals, despite its limitations. Indeed, the PMF is a conceptual framework that allows an understanding of the negative effects of stress on individual psychological dimensions.

Our findings suggest that, unlike in gay men, parenting desire might be influenced by stigmatizing episodes in lesbians, and that this effect is partly explained by the action of sexual orientation concealment. Furthermore, although internalized heterosexism was not a significant mediator between prejudice events and parenting desire, it did increase the level of parenting desire in lesbians, probably because internalizing negative societal values about being lesbian leads to conforming to social expectations that tend to equate women to mothers. Thus, our results might shed light on some clinical implications above all for lesbians, both if they are single or in couple.

Our data should lead clinicians to pay particular attention to minority stress in clinical settings [56,57], and especially to the detrimental effects that stigma, and in particular proximal stressors (i.e., internalized heterosexism and sexual orientation concealment), might have on parenting desire. To this end, clinicians might assume the function of alleviating the emotional impact that internal stressors might have on individuals’ desire, thoughts, or beliefs, helping clients to unhook their self-representation from the social dialectic that still tends to construct a negative image of the LG population, in particular as parents. This seems particular true for lesbians, who live a greater pressure than gay men to conform to social expectations related to parenthood. Indeed, although we usually interpret parenting desire or plan as a sign of comfort towards the self, due to an inherently generative project, our data suggest that the converse is also possible, as both internalized heterosexism and sexual orientation concealment increase, rather than decrease, parenting desire. This means that parenting desire might also be a sign of internalized heterosexism or sexual orientation concealment, rather than a manifestation of a positive identity. Thus, clinicians should deeply explore internal dynamics related to proximal stressors and be cautious in automatically interpreting parenting desire as a sign of comfort.

Furthermore, as both internalized heterosexism and sexual orientation concealment depends on the social stigma that burdens on LG people, clinicians should help clients to develop or increase awareness of stigma, allowing them to perceive oppression as a societal problem that afflict LG individuals as a class of people, rather than as something that concerns them as specific individuals.

Finally, clinicians should also consider group approach as an alternative to individual or couple approach, as the group activates mirroring processes that productively allow participants to reshape self-image [58] and increase the self-empowerment processes [59]. Indeed, LG individuals or couples experiencing high levels of internalized heterosexism might negotiate their negative self-image with peers, ameliorating the impact of stigma on health. In the same vein, the group might also be helpful for LG individuals or couples who tend to conceal their sexual orientation to family, friends, or colleagues. Therefore, sharing one’s own identity to others living similar experiences in a protected and secure setting might allow an elaboration of feelings such as shame and self-hatred, thus facilitating identity or couple affirmation processes. In turn, as suggested by our findings, this clinical work could lighten the weight of social expectations related to parenthood, in particular in women.

## 5. Conclusions

This study provided support for applying the PMF to parenthood dimensions—in particular, parenting desire—in childless LG individuals. Indeed, notwithstanding the limitations of the research, and the nonconfirmation of all of the hypotheses, this study sheds light on the psychological processes that cause stress to increase or decrease the desire to become a parent. Future studies should thoroughly apply the PMF to parenthood dimensions, in order to explore the role of both general psychological processes (e.g., interpersonal problems or emotion dysregulation) and protective factors (e.g., resilience or community connectedness).

## Figures and Tables

**Table 1:** Sociodemographic characteristics of participants (*N* = 290).

Characteristics	Total (*N* = 290) *n* (%) or M ± SD	Lesbians (*n* = 120) *n* (%) or M ± SD	Gay men (*n* = 170) *n* (%) or M ± SD	*p*-Value
Age	31.10 ± 8.69	28.25 ± 6.14	33.11 ± 9.64	<0.001
Education				0.028
≤ High school	116 (40)	57 (47.5)	59 (34.7)	
≥ College	174 (60)	63 (52.5)	111 (65.3)	
Political orientation *				0.061
Left-wing	226 (77.9)	87 (72.5)	139 (81.8)	
Centrist	64 (22.1)	33 (27.5)	31 (18.2)	
Stable partner				0.967
Yes	172 (59.3)	71 (59.2)	101 (59.4)	
No	118 (40.7)	49 (40.8)	69 (40.6)	

Note: M = Mean; SD = Standard Deviation; Group differences in age were assessed through the Student’s t-test; Group differences in all other characteristics were assessed through the χ^2^ test. * No participant declared to be right-wing.

**Table 2:** Descriptive statistics and bivariate correlations between minority stressors, parenting desire, and sociodemographic characteristics.

	1	2	3	4	5	6	7	8
1. Prejudice events	-	−0.04	0.12	0.05	−0.14	−0.07	−0.04	−0.02
2. Internalized heterosexism	0.18 *	-	−0.39 ***	0.04	−0.22 **	−0.12	−0.03	0.21 **
3. Sexual orientation concealment	−0.01	−0.45 ***	-	0.07	0.21 **	−0.02	−0.13	−0.20 *
4. Parenting desire	−0.16	0.18 *	0.14	-	−0.15 *	−0.01	−0.08	−0.08
5. Age	−0.23 *	−0.24 **	0.19 *	−0.12	-	0.07	−0.09	−0.15
6. Education (≤ High school)	−0.06	−0.13	0.09	−0.08	0.38 ***	-	−0.04	−0.13
7. Political orientation (left-wing)	−0.09	−0.05	−0.05	0.04	−0.10	−0.16	-	−0.14
8. Stable partner (yes)	0.09	0.12	−0.26 **	−0.20 *	0.01	0.01	0.06	-

Note: Lesbians scores are below diagonal and gay men scores are above diagonal;*** *p* < 0.001; ** *p* < 0.01; * *p* < 0.05.

**Table 3:** Test of the moderated mediation effect of prejudice events on parenting desire through sexual orientation concealment and internalized heterosexism.

Predictors	Sexual Orientation Concealment	Internalized Heterosexism	Parenting Desire
	B	B	B
Age	0.03 ***	−0.01 **	−0.04 **
Education (≤High school)	−0.04	−0.08	−0.14
Political orientation (left-wing)	0.30	0.06	0.30
Stable partner (yes)	−0.56 ***	0.15 **	−0.62 *
Prejudice events	0.27 *	0.02	0.05
Internalized heterosexism			0.24
Sexual orientation concealment			0.12
Prejudice events X gender			−1.23 *
Internalized heterosexism X gender			2.17 *
Sexual orientation concealment X gender			0.69 *
R ^2^	0.11	0.07	
−2LogLikelihood			292.07 Δ*χ*^2^ (11) = 32.98, *p* = 0.005

Note: Unstandardized coefficients. * *p* ≤ 0.05, ** *p* ≤ 0.01, *** *p* ≤ 0.001.

**Table 4:** Indirect effects of prejudice events on parenting desire through sexual orientation concealment and internalized heterosexism conditional on gender.

	Moderated Mediation Index	
Mediators	B	95% C.I.
Sexual orientation concealment	0.19	0.001, 0.63
Internalized heterosexism	0.05	−0.20, 0.40
